# Prévalence des dyslipidémies au laboratoire de biochimie de l'Hôpital Joseph Ravoahangy-Andrianavalona à Antananarivo, Madagascar

**DOI:** 10.11604/pamj.2026.53.130.49016

**Published:** 2026-03-16

**Authors:** Faralahy Harisolofo Rakotonjafiniarivo, Mahefa Soja Rakotomalala, Tokinomenjanahary Antsonantenaina, Miora Koloina Ranaivosoa

**Affiliations:** 1 Laboratoire de Biochimie du Centre Hospitalier Universitaire Joseph Ravoahangy-Andrianavalona, Antananarivo, Madagascar,; 2 Faculté de Médecine de l'Université d'Antananarivo, Antananarivo, Madagascar

**Keywords:** Dyslipidémie, Madagascar, prévalence

## Aux éditeurs du Pan African Medical Journal

Les dyslipidémies demeurent un réel problème de santé publique. Leur prévalence est variable allant de 30,3% en Asie du Sud-Est à 47,7% en Amérique et à 53,7% en Europe [1]. Dans certaines régions de l'Afrique, la prévalence de la dyslipidémie dépasse 60% [2,3]. A Madagascar, les données sur les facteurs de risque de maladies cardiovasculaires sont rares [4,5]. La réalisation d'un bilan lipidique est une étape dans la stratégie de prévention de ces maladies. Le présent travail a pour objectif d'évaluer la prévalence des dyslipidémies chez des patients reçus au laboratoire de Biochimie du Centre Hospitalier Universitaire Joseph Ravoahangy Andrianavalona (CHU-JRA), situé dans la capitale de Madagascar, pendant 3 ans.

Une étude transversale à visée descriptive a été réalisée portant sur 1572 patients âgés reçus au laboratoire de biochimie du CHU-JRA, situé dans la capitale de Madagascar durant 3 ans, allant de janvier 2022 à décembre 2024. Les patients ayant au moins un paramètre du bilan lipidique ont été inclus dans la présente étude. Les bilans lipidiques ont été dosés sur l'automate BS-300® (*Mindray Medical International Limited China*) par méthodes enzymatiques. Les dyslipidémies ont été définies selon les critères du NCEP (*The National Cholesterol Education Program*). Hypercholestérolémie (cholestérol total > 2g/L soit 5,16 mmol/L), HypoHDLémie (cholestérol HDL <0,4 g/L soit 1,0 mmol/L), HyperLDLémie (Cholestérol LDL > 1,3 g/L soit 3,35 mmol/L), Hypertriglycéridémie (Triglycéride >1,5 g/L soit 1,71 mmol/L), Hyperlipidémie mixte (Cholestérol LDL > 2g/L et Triglycéride > 1,5g/L). Les données ont été collectées sur Excel 2013 à partir du logiciel interne du laboratoire pour les validations des résultats, et analysées par le logiciel Epi Info 7 (version 7.1.0, CDC Atlanta). Les tests statistiques ont été effectués en utilisant le test de khi-deux avec un seuil de significativité de 0,05.

**Figure 1:**
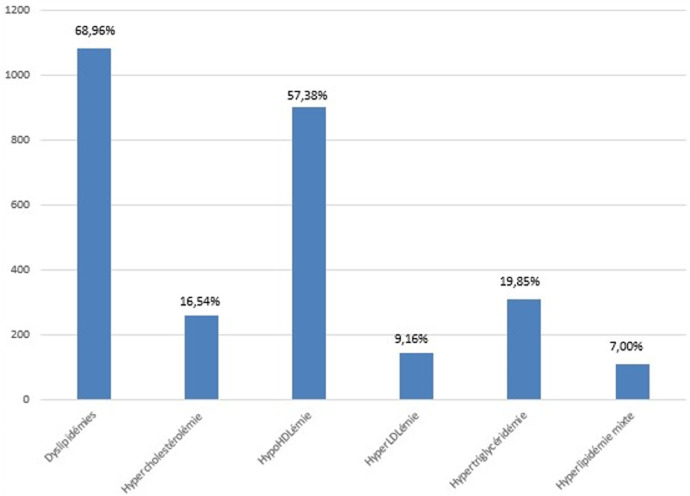
prévalence des dyslipidémies

Le Tableau 1 résume les caractéristiques de la population d'étude. On note une prédominance féminine avec un sex ratio de 0,87. La tranche d'âge de 40 à 59 ans est la plus représentée (45,04%) et 48,66% des patients étaient hospitalisés dans le CHU-JRA. La prévalence des dyslipidémies dans la population d'étude pendant la période étudiée est de 68,96%. Les prévalences de l'hypercholestérolémie, l'hypoHDLémie, l'hyperLDLémie, l'hypertriglycéridémie et l'hyperlipidémie mixte étaient respectivement de 16,54%; 57,38%; 9,16%; 19,85% et 7,00% (Figure 1). Seules 33,06% des demandes d'examen comportaient une information clinique et parmi les renseignements notés, l'hypertension artérielle (HTA) était majoritaire, suivie des complications vasculaires et du diabète. La répartition des dyslipidémies en fonction de l'âge montre une exposition plus marquée des sujets entre 40 et 59 ans. Les dyslipidémies touchent davantage le genre féminin. Les hypercholestérolémie, l'hyperLDLémie et l'hyperlipidémie mixte sont prédominantes chez les femmes (respectivement p= 0,005, p=0,04 et p=0,02). L'hypoHDLémie est plus prédominante chez les hommes (p <0,01).

**Tableau 1:** caractéristiques sociodémographiques de la population d'étude

Variables	Total n (%)	Dyslipidémies	
		**Oui** n (%)	**Non** n (%)
**Genre**			
Masculin	734 (46,69)	544 (74,11)	190 (25,89)
Féminin	838 (53,31)	542 (64,67)	296 (35,33)
**Sex ratio**	0,87		
**Age (année)**			
0-39	283 (18,00)	184 (65,02)	99 (34,98)
40-59	708 (45.04)	500 (70,62)	208 (29,38)
≥60	581 (36.96)	402 (69,20)	179 (30,80)
**Provenance**			
Hospitalisés	765 (48,66)	549 (71,76)	216 (28,24)
Externes	807 (51,34)	537 (66,54)	270 (33,46)
**Informations cliniques**			
HTA	423 (26,91)	277 (65,48)	146 (34,52)
Diabète	42 (2,67)	27 (64,29)	15 (35,71)
Obésité́	2 (0,12)	0 (0,00)	2 (100)
Complications cardio-vasculaires	64 (4,07)	48 (75,00)	16 (25,00)
Autres	1024 (65,14)	727 (70,80)	297 (29,00)
Absence d'information clinique	8 (0,51)	2 (25,00)	6 (75,00)

Les dyslipidémies sont des facteurs de risque de maladies cardiovasculaires liées à l'athérosclérose [6,7]. Ce travail, qui avait pour objectif d'évaluer la prévalence des dyslipidémies chez des patients reçus au laboratoire de Biochimie d'un hôpital de la capitale de Madagascar a retrouvé un taux élevé de 68,96%. Cette prévalence élevée est en augmentation par rapport à une étude réalisée dans un service de réanimation à Madagascar en 2015 [5], mais superposable à celle réalisée dans une région côtière malgache entre 2017 et 2018 [4]. La prévalence des dyslipidémies retrouvée dans cette étude corrobore avec celles retrouvées par des études réalisées au Sénégal [2] et dans la région de l'Afrique de l'Est [3] avec des prévalences respectives de 61,3% et 60,7%. Dans cette étude, l'hypoHDLémie est la plus fréquente des dyslipidémies (57,38%) suivie de l'hypertriglycéridémie (19,85%).

Par contre, des différences ont été retrouvées avec certains auteurs qui ont déterminé la prédominance d'une hypercholestérolémie [4,5]. Concernant l'âge, cette étude a montré que la tranche d'âge de 40 à 59 ans était majoritaire. Ce résultat concorde avec une étude réalisée au Sénégal en 2016 [8]. Une prédominance féminine a été retrouvée pour les dyslipidémies en général et pour l'hypercholestérolémie, l'hyperLDLémie et l'hyperlipidémie mixte en particulier. D'autres auteurs ont mis en évidence une hétérogénéité des dyslipidémies en fonction du genre [9,10].

## Conclusion

cette étude a déterminé une forte prévalence des dyslipidémies dans un milieu hospitalier à Madagascar. Le manque de données cliniques ne permettant pas de spécifier la prise de traitements hypolipémiants pourrait être un biais d'information. De plus larges études sur les facteurs de risque cardiovasculaire spécifiques à la population malgache sont nécessaires pour adapter les stratégies de prévention et de traitement de ces pathologies.
